# *N*-(3-Methyl­phen­yl)succinamic acid

**DOI:** 10.1107/S1600536810001480

**Published:** 2010-01-16

**Authors:** B. Thimme Gowda, Sabine Foro, B. S. Saraswathi, Hartmut Fuess

**Affiliations:** a Department of Chemistry, Mangalore University, Mangalagangotri 574 199, Mangalore, India; b Institute of Materials Science, Darmstadt University of Technology, Petersenstrasse 23, D-64287 Darmstadt, Germany

## Abstract

In the crystal structure of the title compound, C_11_H_13_NO_3_, the conformations of the N—H and C=O bonds in the amide segment are *anti* to each other, and that of the amide H atom is *anti* to the *meta*-methyl group in the benzene ring. Furthermore, the conformations of the amide oxygen and the carbonyl O atom of the acid segment are also *anti* to the adjacent –CH_2_ groups. The C=O and O—H bonds of the acid group are *syn* to each other. In the crystal, the mol­ecules are packed into infinite chains through inter­molecular N—H⋯O and O—H⋯O hydrogen bonds.

## Related literature

For our studies on the effect of ring and side-chain substitutions on the solid-state geometry of anilides, see: Gowda *et al.* (2007 ▶; 2009**a* ▶,b* ▶). For the modes of inter­linking carboxylic acids by hydrogen bonds, see: Leiserowitz (1976 ▶). For the packing of mol­ecules involving dimeric hydrogen-bonded association of each carboxyl group with a centrosymmetrically related neighbor, see: Jagannathan *et al.* (1994 ▶).
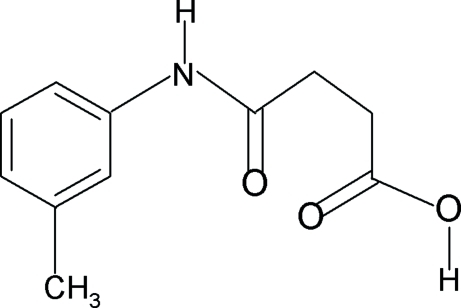


## Experimental

### 

#### Crystal data

C_11_H_13_NO_3_
*M* *_r_* = 207.22
Orthorhombic, 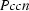

*a* = 12.0661 (8) Å
*b* = 20.220 (1) Å
*c* = 8.9398 (5) Å
*V* = 2181.1 (2) Å^3^
*Z* = 8
Mo *K*α radiation
μ = 0.09 mm^−1^
*T* = 299 K
0.44 × 0.34 × 0.22 mm

#### Data collection

Oxford Diffraction Xcalibur diffractometer with a Sapphire CCD detector
Absorption correction: multi-scan (*CrysAlis RED*; Oxford Diffraction, 2009 ▶) *T*_min_ = 0.961, *T*_max_ = 0.980
9274 measured reflections
2228 independent reflections
1772 reflections with *I* > 2σ(*I*)
*R*_int_ = 0.019

#### Refinement

*R*[*F* ^2^ > 2σ(*F* ^2^)] = 0.045
*wR*(*F* ^2^) = 0.121
*S* = 1.05
2228 reflections
167 parameters
1 restraint
H atoms treated by a mixture of independent and constrained refinement
Δρ_max_ = 0.18 e Å^−3^
Δρ_min_ = −0.21 e Å^−3^

### 

Data collection: *CrysAlis CCD* (Oxford Diffraction, 2009 ▶); cell refinement: *CrysAlis RED* (Oxford Diffraction, 2009 ▶); data reduction: *CrysAlis RED*; program(s) used to solve structure: *SHELXS97* (Sheldrick, 2008 ▶); program(s) used to refine structure: *SHELXL97* (Sheldrick, 2008 ▶); molecular graphics: *PLATON* (Spek, 2009 ▶); software used to prepare material for publication: *SHELXL97*.

## Supplementary Material

Crystal structure: contains datablocks I, global. DOI: 10.1107/S1600536810001480/bq2189sup1.cif

Structure factors: contains datablocks I. DOI: 10.1107/S1600536810001480/bq2189Isup2.hkl

Additional supplementary materials:  crystallographic information; 3D view; checkCIF report

## Figures and Tables

**Table 1:** Hydrogen-bond geometry (Å, °)

*D*—H⋯*A*	*D*—H	H⋯*A*	*D*⋯*A*	*D*—H⋯*A*
N1—H1*N*⋯O1^i^	0.863 (19)	2.02 (2)	2.8597 (17)	163.5 (16)
O3—H3*O*⋯O2^ii^	0.83 (1)	1.82 (1)	2.6542 (18)	177 (2)

Symmetry codes: (i) 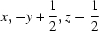; (ii) 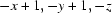.

**Fig. 1.**
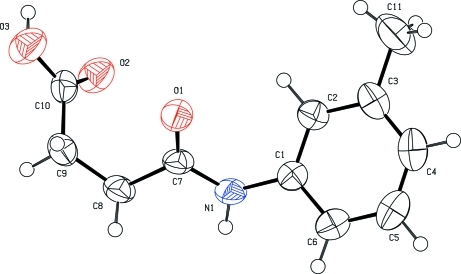
Molecular structure of the title compound, showing the atom labelling scheme. The displacement ellipsoids are drawn at the 50% probability level.

**Fig. 2.**
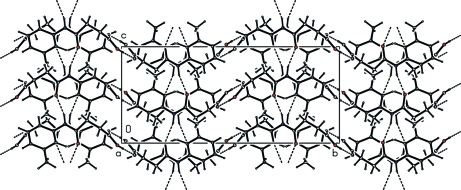
Molecular packing of the title compound with hydrogen bonding shown as dashed lines.

## supplementary crystallographic information

### Comment

As a part of studying the effect of ring and side chain substitutions on the solid state geometry of anilides (Gowda *et al.*, 2007; 2009*a,b*), we report herein the crystal structure of *N*-(3-methylphenyl)succinamic acid (I). The conformations of N—H and C=O bonds in the amide segment are *anti* to each other. But the conformation of the amide oxygen and the carbonyl oxygen of the acid segment are *syn* to each other, contrary to the *anti* conformation observed in *N*-(4-Chlorophenyl)succinamic acid (II) (Gowda *et al.*, 2009*a*) and *N*-(2-chlorophenyl)- succinamic acid (III)(Gowda *et al.*, 2009*b*). Further, the conformation of both the C=O bonds are *anti* to the H atoms of their adjacent –CH_2_ groups (Fig. 1) and the C=O and O—H bonds of the acid group are in *syn* position to each other, similar to that observed in (II) and (III).

The conformation of the amide hydrogen is *anti* to the *meta*- methyl group in the benzene ring, contrary to the *syn* conformation observed between the amide hydrogen and the *ortho*-Cl in (III).

The N—H···O and O—H···O intermolecular hydrogen bonds pack the molecules into infinite chains in the structure (Table 1, Fig.2).

The modes of interlinking carboxylic acids by hydrogen bonds is described elsewhere (Leiserowitz, 1976). The packing of molecules involving dimeric hydrogen bonded association of each carboxyl group with a centrosymmetrically related neighbor has also been observed (Jagannathan *et al.*, 1994).

### Experimental

The solution of succinic anhydride (0.01 mole) in toluene (25 ml) was treated dropwise with the solution of *m*-toluidine (0.01 mole) also in toluene (20 ml) with constant stirring. The resulting mixture was stirred for about one h and set aside for an additional hour at room temperature for completion of the reaction. The mixture was then treated with dilute hydrochloric acid to remove the unreacted *m*-toluidine. The resultant solid *N*-(3-methylphenyl)- succinamic acid was filtered under suction and washed thoroughly with water to remove the unreacted succinic anhydride and succinic acid. It was recrystallized to constant melting point from ethanol.

The purity of the compound was checked by elemental analysis and characterized by its infrared and NMR spectra. The rod like colorless single crystals used in X-ray diffraction studies were grown in ethanolic solution by slow evaporation at room temperature.

### Refinement

The H atoms of the CH_3_ group were positioned with idealized geometry using a riding model with C—H = 0.96 Å. The other H atoms were located in a difference map and their positions refined [N—H = 0.86 (2) %A, C—H = 0.93 (2)–1.01 (2) Å.], while the H atom of the OH group was later restrained to the distance O—H = 0.82 (1) Å. All H atoms were refined with isotropic displacement parameters (set to 1.2 times of the *U*_eq_ of the parent atom).

### Crystal data

**Table:**

C_11_H_13_NO_3_	*F*(000) = 880
*M**_r_* = 207.22	*D*_x_ = 1.262 Mg m^−^^3^
Orthorhombic, *P**c**c**n*	Mo *K*α radiation, λ = 0.71073 Å
Hall symbol: -P 2ab 2ac	Cell parameters from 4700 reflections
*a* = 12.0661 (8) Å	θ = 2.5–27.8°
*b* = 20.220 (1) Å	µ = 0.09 mm^−^^1^
*c* = 8.9398 (5) Å	*T* = 299 K
*V* = 2181.1 (2) Å^3^	Rod, colourless
*Z* = 8	0.44 × 0.34 × 0.22 mm

### Data collection

**Table:**

Oxford Diffraction Xcalibur diffractometer with a Sapphire CCD detector	2228 independent reflections
Radiation source: fine-focus sealed tube	1772 reflections with *I* > 2σ(*I*)
graphite	*R*_int_ = 0.019
Rotation method data acquisition using ω and φ scans.	θ_max_ = 26.4°, θ_min_ = 3.0°
Absorption correction: multi-scan (*CrysAlis RED*; Oxford Diffraction, 2009)	*h* = −15→13
*T*_min_ = 0.961, *T*_max_ = 0.980	*k* = −25→25
9274 measured reflections	*l* = −9→11

### Refinement

**Table:**

Refinement on *F*^2^	Primary atom site location: structure-invariant direct methods
Least-squares matrix: full	Secondary atom site location: difference Fourier map
*R*[*F*^2^ > 2σ(*F*^2^)] = 0.045	Hydrogen site location: inferred from neighbouring sites
*wR*(*F*^2^) = 0.121	H atoms treated by a mixture of independent and constrained refinement
*S* = 1.05	*w* = 1/[σ^2^(*F*_o_^2^) + (0.0567*P*)^2^ + 0.6529*P*] where *P* = (*F*_o_^2^ + 2*F*_c_^2^)/3
2228 reflections	(Δ/σ)_max_ = 0.029
167 parameters	Δρ_max_ = 0.18 e Å^−^^3^
1 restraint	Δρ_min_ = −0.21 e Å^−^^3^

### Special details

**Table:**

Experimental. CrysAlis RED (Oxford Diffraction, 2009) Empirical absorption correction using spherical harmonics, implemented in SCALE3 ABSPACK scaling algorithm.
Geometry. All e.s.d.'s (except the e.s.d. in the dihedral angle between two l.s. planes) are estimated using the full covariance matrix. The cell e.s.d.'s are taken into account individually in the estimation of e.s.d.'s in distances, angles and torsion angles; correlations between e.s.d.'s in cell parameters are only used when they are defined by crystal symmetry. An approximate (isotropic) treatment of cell e.s.d.'s is used for estimating e.s.d.'s involving l.s. planes.
Refinement. Refinement of *F*^2^ against ALL reflections. The weighted *R*-factor *wR* and goodness of fit *S* are based on *F*^2^, conventional *R*-factors *R* are based on *F*, with *F* set to zero for negative *F*^2^. The threshold expression of *F*^2^ > σ(*F*^2^) is used only for calculating *R*-factors(gt) *etc*. and is not relevant to the choice of reflections for refinement. *R*-factors based on *F*^2^ are statistically about twice as large as those based on *F*, and *R*- factors based on ALL data will be even larger.

### Fractional atomic coordinates and isotropic or equivalent isotropic displacement parameters (Å^2^)

**Table:**

	*x*	*y*	*z*	*U*_iso_*/*U*_eq_	
O1	0.60319 (12)	0.30046 (6)	0.00527 (12)	0.0586 (4)	
O2	0.51371 (10)	0.44029 (6)	−0.12328 (15)	0.0604 (4)	
O3	0.64575 (11)	0.48088 (7)	0.02306 (17)	0.0681 (4)	
H3O	0.5948 (14)	0.5046 (10)	0.056 (3)	0.082*	
N1	0.55127 (12)	0.24064 (6)	−0.19678 (14)	0.0449 (3)	
H1N	0.5651 (14)	0.2366 (9)	−0.291 (2)	0.054*	
C1	0.49463 (12)	0.18766 (7)	−0.12564 (16)	0.0398 (4)	
C2	0.42443 (13)	0.19787 (8)	−0.00544 (18)	0.0456 (4)	
H2	0.4131 (14)	0.2440 (10)	0.0343 (19)	0.055*	
C3	0.37093 (14)	0.14509 (9)	0.0632 (2)	0.0541 (4)	
C4	0.38770 (17)	0.08225 (9)	0.0064 (2)	0.0617 (5)	
H4	0.3523 (18)	0.0467 (10)	0.052 (2)	0.074*	
C5	0.45474 (17)	0.07211 (9)	−0.1154 (2)	0.0627 (5)	
H5	0.4654 (17)	0.0287 (11)	−0.156 (2)	0.075*	
C6	0.50913 (15)	0.12444 (8)	−0.1827 (2)	0.0505 (4)	
H6	0.5552 (15)	0.1191 (9)	−0.270 (2)	0.061*	
C7	0.60056 (13)	0.29228 (7)	−0.13003 (16)	0.0401 (4)	
C8	0.65448 (16)	0.34114 (8)	−0.23546 (18)	0.0470 (4)	
H8A	0.5993 (15)	0.3536 (8)	−0.311 (2)	0.056*	
H8B	0.7113 (15)	0.3183 (8)	−0.285 (2)	0.056*	
C9	0.69866 (15)	0.40119 (9)	−0.1542 (2)	0.0514 (4)	
H9A	0.7504 (17)	0.3894 (9)	−0.078 (2)	0.062*	
H9B	0.7338 (16)	0.4291 (9)	−0.223 (2)	0.062*	
C10	0.61046 (13)	0.44199 (7)	−0.08284 (18)	0.0449 (4)	
C11	0.2978 (2)	0.15574 (12)	0.1975 (3)	0.0901 (8)	
H11A	0.2219	0.1582	0.1660	0.108*	
H11B	0.3183	0.1962	0.2462	0.108*	
H11C	0.3067	0.1195	0.2658	0.108*	

### Atomic displacement parameters (Å^2^)

**Table:**

	*U*^11^	*U*^22^	*U*^33^	*U*^12^	*U*^13^	*U*^23^
O1	0.0916 (10)	0.0551 (7)	0.0292 (6)	−0.0188 (6)	0.0069 (6)	−0.0009 (5)
O2	0.0537 (7)	0.0579 (7)	0.0698 (8)	0.0037 (6)	−0.0091 (6)	−0.0140 (6)
O3	0.0554 (8)	0.0659 (8)	0.0831 (10)	0.0010 (6)	−0.0092 (7)	−0.0255 (7)
N1	0.0613 (8)	0.0467 (7)	0.0267 (6)	−0.0016 (6)	0.0071 (6)	−0.0018 (5)
C1	0.0431 (8)	0.0422 (8)	0.0342 (7)	0.0017 (6)	−0.0055 (6)	0.0014 (6)
C2	0.0459 (9)	0.0474 (8)	0.0436 (9)	0.0020 (7)	0.0020 (7)	0.0015 (7)
C3	0.0424 (9)	0.0623 (10)	0.0577 (10)	−0.0037 (7)	0.0012 (8)	0.0128 (8)
C4	0.0574 (11)	0.0524 (10)	0.0752 (13)	−0.0111 (8)	−0.0087 (10)	0.0160 (9)
C5	0.0740 (13)	0.0417 (9)	0.0724 (13)	−0.0008 (8)	−0.0147 (11)	−0.0031 (8)
C6	0.0569 (10)	0.0483 (9)	0.0463 (9)	0.0059 (7)	−0.0047 (8)	−0.0042 (7)
C7	0.0499 (9)	0.0410 (7)	0.0294 (7)	0.0048 (6)	0.0062 (6)	0.0010 (6)
C8	0.0560 (10)	0.0482 (9)	0.0367 (8)	0.0014 (7)	0.0130 (8)	0.0038 (7)
C9	0.0490 (9)	0.0527 (9)	0.0525 (10)	−0.0063 (8)	0.0105 (8)	0.0061 (8)
C10	0.0493 (9)	0.0380 (7)	0.0473 (9)	−0.0073 (6)	−0.0001 (7)	0.0057 (6)
C11	0.0777 (14)	0.0925 (16)	0.0999 (18)	−0.0039 (12)	0.0402 (13)	0.0214 (14)

### Geometric parameters (Å, °)

**Table:**

O1—C7	1.2212 (17)	C4—H4	0.93 (2)
O2—C10	1.2226 (19)	C5—C6	1.383 (3)
O3—C10	1.302 (2)	C5—H5	0.96 (2)
O3—H3O	0.832 (10)	C6—H6	0.96 (2)
N1—C7	1.3416 (19)	C7—C8	1.513 (2)
N1—C1	1.4210 (19)	C8—C9	1.512 (2)
N1—H1N	0.863 (19)	C8—H8A	0.983 (19)
C1—C2	1.384 (2)	C8—H8B	0.940 (19)
C1—C6	1.387 (2)	C9—C10	1.490 (2)
C2—C3	1.390 (2)	C9—H9A	0.95 (2)
C2—H2	1.008 (19)	C9—H9B	0.94 (2)
C3—C4	1.383 (3)	C11—H11A	0.9600
C3—C11	1.505 (3)	C11—H11B	0.9600
C4—C5	1.372 (3)	C11—H11C	0.9600
			
C10—O3—H3O	111.1 (16)	O1—C7—C8	121.17 (14)
C7—N1—C1	126.93 (12)	N1—C7—C8	114.95 (13)
C7—N1—H1N	115.0 (12)	C9—C8—C7	112.13 (13)
C1—N1—H1N	117.2 (12)	C9—C8—H8A	111.4 (10)
C2—C1—C6	120.02 (15)	C7—C8—H8A	107.8 (10)
C2—C1—N1	121.97 (13)	C9—C8—H8B	111.4 (11)
C6—C1—N1	117.99 (14)	C7—C8—H8B	106.8 (11)
C1—C2—C3	120.83 (15)	H8A—C8—H8B	107.1 (15)
C1—C2—H2	119.6 (10)	C10—C9—C8	113.49 (15)
C3—C2—H2	119.5 (10)	C10—C9—H9A	107.6 (12)
C4—C3—C2	118.37 (17)	C8—C9—H9A	111.9 (11)
C4—C3—C11	120.66 (17)	C10—C9—H9B	105.8 (11)
C2—C3—C11	120.96 (18)	C8—C9—H9B	109.0 (11)
C5—C4—C3	121.00 (17)	H9A—C9—H9B	108.8 (16)
C5—C4—H4	120.2 (13)	O2—C10—O3	122.97 (15)
C3—C4—H4	118.8 (13)	O2—C10—C9	122.68 (15)
C4—C5—C6	120.73 (17)	O3—C10—C9	114.33 (15)
C4—C5—H5	120.9 (13)	C3—C11—H11A	109.5
C6—C5—H5	118.4 (13)	C3—C11—H11B	109.5
C5—C6—C1	119.01 (17)	H11A—C11—H11B	109.5
C5—C6—H6	122.7 (11)	C3—C11—H11C	109.5
C1—C6—H6	118.2 (11)	H11A—C11—H11C	109.5
O1—C7—N1	123.88 (14)	H11B—C11—H11C	109.5
			
C7—N1—C1—C2	41.4 (2)	C2—C1—C6—C5	−1.6 (2)
C7—N1—C1—C6	−140.25 (16)	N1—C1—C6—C5	−179.93 (15)
C6—C1—C2—C3	2.4 (2)	C1—N1—C7—O1	0.1 (3)
N1—C1—C2—C3	−179.29 (14)	C1—N1—C7—C8	−179.98 (14)
C1—C2—C3—C4	−1.4 (3)	O1—C7—C8—C9	−5.6 (2)
C1—C2—C3—C11	177.74 (18)	N1—C7—C8—C9	174.51 (14)
C2—C3—C4—C5	−0.5 (3)	C7—C8—C9—C10	−64.8 (2)
C11—C3—C4—C5	−179.61 (19)	C8—C9—C10—O2	−21.9 (2)
C3—C4—C5—C6	1.3 (3)	C8—C9—C10—O3	160.00 (15)
C4—C5—C6—C1	−0.3 (3)		

### Hydrogen-bond geometry (Å, °)

**Table:**

*D*—H···*A*	*D*—H	H···*A*	*D*···*A*	*D*—H···*A*
N1—H1N···O1^i^	0.863 (19)	2.02 (2)	2.8597 (17)	163.5 (16)
O3—H3O···O2^ii^	0.83 (1)	1.82 (1)	2.6542 (18)	177 (2)

Symmetry codes: (i) *x*, −*y*+1/2, *z*−1/2; (ii) −*x*+1, −*y*+1, −*z*.
